# Safranal as a safe compound to mice immune system

**Published:** 2015

**Authors:** Bamdad Riahi-Zanjani, Mahdi Balali-Mood, Elaheh Mohammadi, Hassan Badie-Bostan, Bahram Memar, Gholamreza Karimi

**Affiliations:** 1 *Medical Toxicology Research Center, School of Medicine, Mashhad University of Medical Sciences, Mashhad, Iran*; 2 *Medical Toxicology Research Center and Pharmacy School, Mashhad University of Medical Sciences, Mashhad, Iran*; 3 *Cancer Research Center, School of Medicine, Mashhad University of Medical Sciences, Mashhad, Iran*

**Keywords:** *Safranal*, *Immunotoxic effect*, *Cellular immunity*, *Humoral immunity*

## Abstract

**Objectives:**

The aim of the present study was to investigate immunotoxic effect of safranal (SAF), a main component of *Crocus sativus* essential oil, using Balb/c mice.

**Materials and Methods:**

SAF was administered intraperitoneally at doses of 0.1, 0.5 and 1 ml/kg for 3 weeks. Hystopathological examination of spleen and bone marrow, cellularity of spleen, delayed type of hypersensitivity (DTH) response, hemagglutination titer (HA), cytokine production and lymphocyte proliferation assay were studied in various groups of animals.

**Results:**

Spleen cellularity for SAF groups (0.1 ml/kg SAF: 6.68 [± 0.88] × 10^7^, 0.5 ml/kg SAF: 8.16 [± 1.33] × 10^7^, 1 ml/kg SAF: 6.12 [± 0.59] × 10^7^) did not significantly differ as compared to vehicle control (8.52 [± 1.36] × 10^7^; p > 0.05). In addition, SAF at all doses could not produce any signiﬁcant changes in hematological parameters, HA titer, DTH and lymphoproliferation responses, as well as in release of cytokines by isolated splenocytes (p > 0.05). Despite a few studies demonstrating some immunomodulatory effects for saffron extract, SAF as a major constituent of saffron did not induce any marked effects in immune system parameters of mice.

**Conclusion:**

Contrary to the toxicological studies which have indicated that SAF is more toxic than other active constituents in saffron stigma, at least it was found to be safe to mice immune system and has no toxicity on humoral and cellular immune responses.

## Introduction

Human body is protected against pathogens such as bacteria and viruses by the immune system. Therefore, it is most important to have a healthy immune apparatus functioning in harmony with other body systems. Conditions suppressing the immune function result in increasing the risks of infection and formation of certain cancers (Bendich, 1993 ▶). As the field of immunotoxicology has matured over the past decades, natural compound have become important candidates for investigation of the immunosuppression/stimulation of widely used natural compounds (Farsam et al., 2011 ▶, Rahnama et al., 2014 ▶, Noori et al., 2010 ▶). Ever since ancient times, the people for treatment of their illness have been looking for drugs in nature. Application of natural medicines was intensified in the world because of their low adverse effects, price and good efficacy in most human illnesses (Hasani-Ranjbar et al., 2009 ▶). *Crocus sativus, *a member of Iridaceae family, commonly known as saffron, has been broadly used in folk medicine (Zargari, 1990 ▶). SAF, a major constituent of *C. sativus* essential oil, is supposed to be the main cause of saffron odor. This compound was discovered around eighty years ago and since then different scientific studies have been performed to evaluate its pharmacological and biological activities (Rezaee and Hosseinzadeh, 2013 ▶). 

SAF which is known as an antioxidant (Assimopoulou et al., 2005 ▶, Kanakis et al., 2007 ▶), is thought to have different pharmacological properties like antidepressant (Hosseinzadeh et al., 2004 ▶), anticonvulsant (Hosseinzadeh and Talebzadeh, 2005 ▶), antitussive (Hosseinzadeh and Ghenaati, 2006 ▶), antihypertensive (Boskabady and Aslani, 2006 ▶), cytotoxic (Abdullaev et al., 2003 ▶), antibiotic (Pintado et al., 2011 ▶), gasteroprotective (Kianbakht and Mozaffari, 2009 ▶) and anti-carcinogenic effects (Escribano et al., 1996 ▶). These promising properties of SAF propose its presence as a therapeutic agent in future, although there is a great need for further clinical trials and toxicological studies such as immunotoxicity. Because of high significance of having a perfect immune system, lack of information about immunotoxicity of SAF, and existing of studies suggesting higher toxicity of SAF in comparison to other components of saffron plant (Ziaee et al., 2014 ▶), we aimed at evaluating subacute effects of SAF on immune system parameters in Balb/c mice.

## Materials and Methods

**Animals**

Male Balb/c inbred mice (6-8 weeks old) were purchased from Razi Vaccine and Serum Research Institute, Mashhad, Iran. Animals were acclimatized to laboratory conditions for at least one week prior to use. Mice were housed in polystyrene cages *ad libitum* access to food and water with an ambient temperature of 20–25 ºC under a 12 h light/dark. All animal experiments were carried out in accordance with Mashhad University of Medical Sciences, Ethical Committee acts.

**Chemicals**

Phytohemagglutinin-A (PHA), cyclophosphamide and safranal (with purity of ≥ 88%) were purchased from Sigma (UK). Fetal bovine serum and RPMI-1640 medium were purchased from Gibco (UK). SRBC were obtained from Razi Institute (Mashhad, Iran). Sandwich ELISA kits for quantitation of IFNγ and IL4 were purchased from ebioscience Company.

**Doses and exposure schedules**

Five groups of mice (six mice per group) were treated by different doses of SAF, positive (cyclophosphamide) and negative (paraffin) controls. Animals in the SAF experimental groups were injected intraperitoneally by suitable volumes of SAF solutions (prepared in paraffin solution) in order to receive 0.1, 0.5 and 1 ml/kg of SAF for 3 weeks (5 days/week). Different mice groups were used for each experiment. Mice in the vehicle control group received only paraffin injections for 3 weeks (5 days/week). Positive control groups received cyclophosphamide at 20 mg/kg/day for 5 days.

**Determination of the hematological parameters**

Blood was collected from the retro-orbital plexus of each mouse before they were sacrificed by cervical dislocation. Blood (0.2 ml) was collected into sterile (K-EDTA) anti-coagulated tubes to permit total WBC (white blood cell) determinations. A blood smear was also prepared, stained with Giemsa dye, and then examined under a light microscope for differential analyses (based on counts of at least 200 cells/slide/mouse) (Riahi et al., 2010 ▶).

**Histopathological examination**

On day 21, groups of mice were sacrificed by cervical dislocation for all histopathological investigations. The spleen of each mouse were then collected and fixed in 10% formalin. Following mounting, 5-μm thick sections of these tissues were stained with Hematoxylin & Eosin (H&E). In addition, the femurs of each mouse were collected and bone marrow smears prepared and stained with H&E. Histopathological changes of organs were then investigated via light microscopy and scored based on the degree of changes present (Neishabouri et al., 2004 ▶)

**Preparation of single-cell suspension and splenocyte enumeration**

Each spleen isolated was placed into a small petri dish including 10 ml RPMI-1640 media supplemented with 10% fetal bovine serum (FBS), 100 U/ml penicillin, 100 µg/ml streptomycin and 2 mM glutamine. The spleen was teased between two frosted slides and the tissue dispersion generated was recovered and then centrifuged at 1200 rpm at 4 ℃for 10 min. The supernatant was removed and the pellet re-suspended in 3 ml of RBC lysing buffer containing 0.83% NH4Cl in 100 mM Tris buffer, pH 7.4 and kept at room temperature for 3 min. The cells were washed three times with the media and suspended into 1 ml of the media containing 10% FBS. Spleen cellularity was determined by using the Neubauer chamber. Viability of cells was performed using the trypan blue exclusion method (Riahi et al., 2010 ▶).

**Hemagglutination assay**

Four days before ending the treatment period (i.e., on Day 17), sets of mice in each regimen were immunized i.p. by 5×10^8^ SRBCs in PBS. Injection of SAF was continued until day 21. At the end of experiment, after providing sera from collected blood samples, aliquots (50 µl) of two-fold dilutions of the sera (in PBS) were challenged with 50 µl of a 2% [v/v] SRBC suspension in a glass tubes. The tubes were incubated at 37 ºC for 4 h and then observed for hemagglutination. The highest dilution giving hemagglutination was considered as the antibody titer (Riahi et al., 2010 ▶).

**Lymphocyte proliferation test**

Lymphocyte proliferation was done as previously described (Mosmann, 1983 ▶). The 100 µl aliquots of the splenocytes at 2×10^6 ^cells/ml were placed into wells of a 96-well microtiter plate. To each well was added either Phytohemagglutinin-A (PHA) at a final concentration of 5 µg/ml or medium only. The cultures were then incubated for 48 h at 37 ºC and 5% CO2 in humid incubator and then cell proliferation was determined by MTT-based assay. Briefly, 15µl of a 5 mg/ml solution of 3-(4,5-diamethyl-2-thiazolyl) 2,5-diphenyl-2H-tetrazolium (MTT); was added to each well and incubated at 37 ºC in CO2 humid incubator for 4 h. The blue formazan precipitate was then dissolved in dimethylsulfoxideand (DMSO) and the optical density in each well was determined at 570 nm by using Stat-Fax™ Elisa Reader. Proliferation index (PI) was calculated as follows:

PI = Absorbance of stimulated cells/Absorbance of unstimulated cells.

**Delayed-type hypersensitivity response (DTH)**

Delayed-type hypersensitivity response (DTH) was determined for all mice groups using the method of Fararjeh et al. (2008) ▶ (Fararjeh et al., 2008 ▶). On the 16th day of the treatment, mice were injected and immunized i.p. with 100 µl of a 1×10^10^ cells/ml solution of SRBCs. Five days after immunization; all animals were again challenged with a booster dose of 1×10^8^/50 µl SRBCs in the left hind footpad. The right hind footpad was injected with the same volume of PBS to serve as trauma control for non-specific swelling. Increase in left footpad volume was determined 24 and 48 h after the challenge with SRBCs and the mean percentage increase in the foot pad thickness was calculated according to the following formula. 

$\frac{\left(\mathrm{Left footpad challenged with SRBC}-\mathrm{Right footpad}\right)\times 100}{\mathrm{Right footpad}}$

**Cytokine production**

For this purpose, after 48 h of splenocyte cultivation in the presence of PHA mitogen (as previously described in Section of Lymphocyte Proliferation Test), supernatants were harvested and kept at ^_^70 ºC until testing. IFNγ and IL-4 levels were determined using commercially available ELISA kits according to the manufacturer’s protocol (Zheng et al., 1998 ▶).

**Statistical analysis**

Data were statistically analyzed using Student’s t-test to determine significant differences in the data of various groups. P values less than 0.05 were considered significant. The values are expressed as means ± SEM.

## Results

**Hematological parameters**

SAF at three doses did not induce any significant differences in absolute numbers of mice white blood cells, neutrophils, lymphocytes and monocytes as compared to vehicle control group. Cyclophosphamide decreased WBC, lymphocytes and monocytes significantly as shown in table 1.

**Spleen cellularity**

As shown in table 1, no significant difference in spleen cellularity was observed among SAF treatment group compared to vehicle control. The positive control also showed no significant change in this experiment (Table 1).

**Histopathological examination**

*Spleen*

Spleen was evaluated for white pulp atrophy (or hyperplasia), red pulp:white pulp ratio, and for the precence of clumps, debris, necrosis, and apoptosis in the white and red pulp regions . In addition, any splenic trabecular disorders were investigated. The analyses revealed that SAF had not significant adverse effect on spleen.

**Table 1:** Effect of subacute exposure to safranal (SAF i.p. for 3 weeks) on mice blood cellularity and splenocyte enumeration.

**Parameter**	**Vehicle control**	**SAF 0.1ml/kg**	**SAF 0.5 ml/kg**	**SAF 1ml/kg**	**Cyclophosphamide**
**Blood WBC (cell/µl)**	9190 ± 388	9250 ± 235	8240 ± 454	8700 ± 498	4750 ± 132***
**Blood neutrophil (cell/µl)**	1976 ± 156	1610 ± 118	1505 ± 153	1949 ± 123	2270 ± 140
**Bood lymphocyte (cell/µl)**	7029 ± 247	7500 ± 209	6555 ± 363	6603 ± 484	2346 ± 205***
**Blood monocyte (cell/µl)**	184 ± 8	140 ± 11	179 ± 14	148 ± 17	133 ± 11**
**Spleen cell content (×10** ^7^ **)**	8.52 ± 1.36	6.68 ± 0.88	8.16 ± 1.33	6.12 ± 0.59	5.77 ± 1.52

Data showed as mean ± SEM.

** P < 0.01 indicates significant changes compared to the vehicle control group.

*** P < 0.001 indicates significant changes compared to the vehicle control group.

*Bone marrow*

Cellularity, the presence and maturation of hematopoietic cell subtypes, as well as the erythroid: myeloid cell ratio, in each bone marrow specimen isolated was evaluated. Using light microscopic examination, no significant pathologic differences were observed among the samples from the different treatment groups.

**Hemagglutination (HA) titer assay**

**Figure1:**
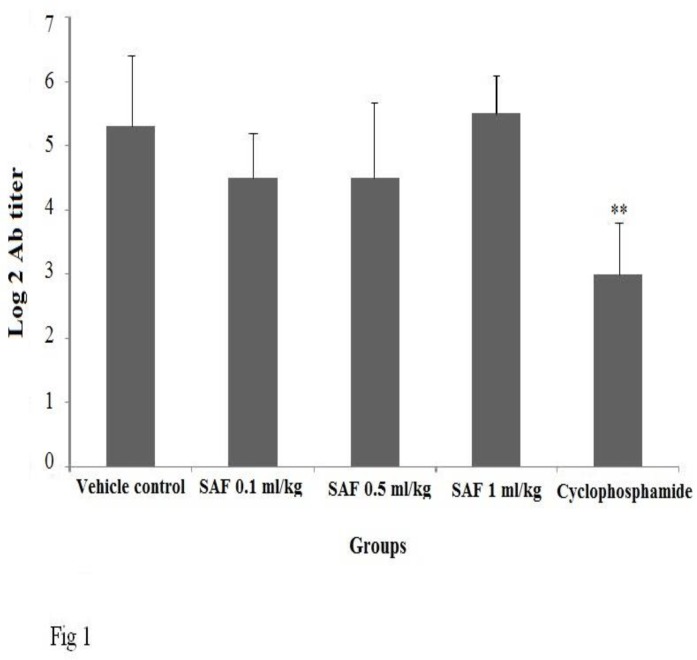
Effect of subacute exposure to safranal (SAF i.p. for 3 weeks) on mice antibody response. Data shown as mean ± SEM. **p<0.01 indicates significant changes compared to the vehicle control group

Serum anti-SRBC titer did not show any significant difference between the SAF treated and negative control group. The positive control significantly (P < 0.01) suppressed production of antibody against SRBCs (Figure 1).

**Proliferative response to PHA**

**Figure 2:**
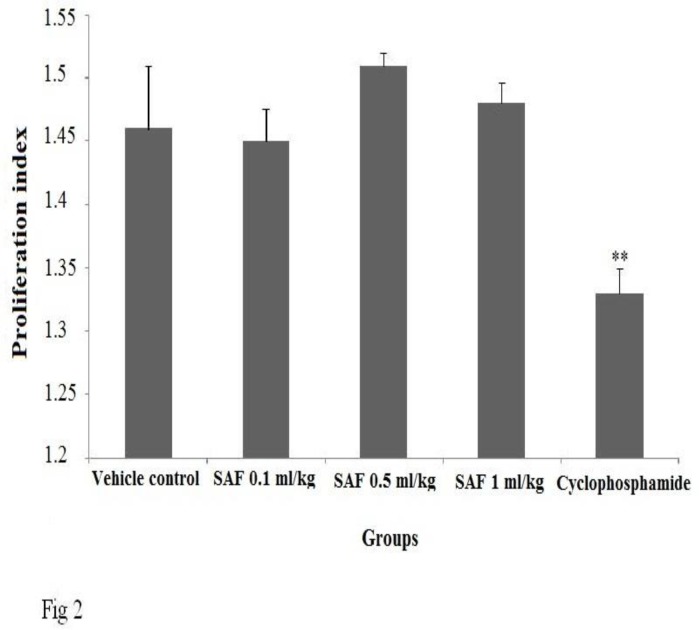
Effect of subacute exposure to safranal (SAF i.p. for 3 weeks) on mice lymphoproliferation response to PHA. Data shown as mean ± SEM. **p<0.01 indicates significant changes compared to the vehicle control group.

SAF at all doses investigated in the study did not show any significant changes in proliferation response. Cyclophosphamide, as positive control, significantly (p<0.01) suppressed the proliferative response (Figure 2).

**Delayed-type hypersensitivity response (DTH)**

**Figure 3:**
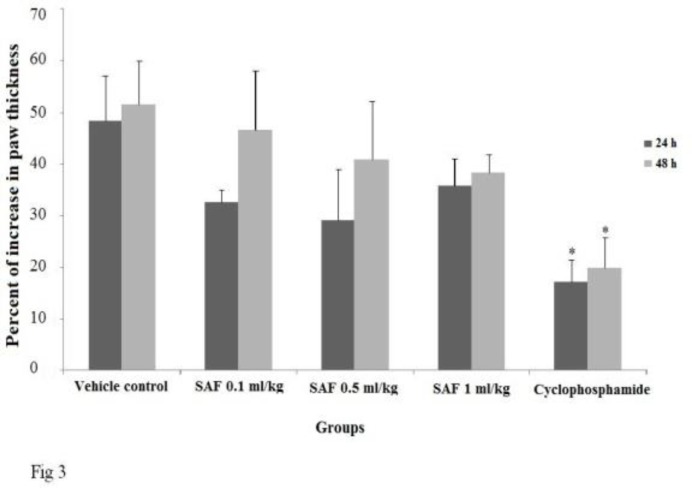
Effect of subacute exposure to safranal (SAF i.p. for 3 weeks) on mice DTH response. Data shown as mean ± SEM. *p<0.05 indicates significant changes compared to the vehicle control group

No significant differences in 24 and 48h-DTH response of SAF treated groups were observed when compared with vehicle control (Figure 3). The positive control group showed significant suppression in DTH response (P < 0.05).

**Cytokine production**

**Figure 4:**
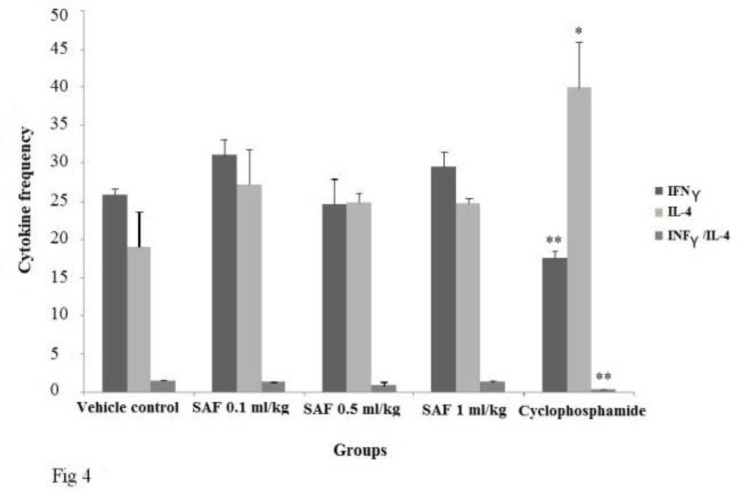
Effect of subacute exposure to safranal (SAF i.p. for 3 weeks) on cytokine produced (pg/ml) by isolated mice splenocytes. IFNγ/IL-4 ratio was also calculated. Data shown as mean ± SEM. *P < 0.05 indicates significant changes compared to the vehicle control group. **P < 0.01 indicates significant changes compared to the vehicle control group

We did not observe any significant difference between the SAF treated and negative control group in levels of cytokine produced by splenocytes (Figure 4). Cyclophosphamide significantly suppressed the production of IFNγ (p<0.01), while IL-4 produced by splenocytes showed a marked elevation in this group (Figure 4). 

## Discussion

Despite the overt use of saffron in food and drug industries, there is no information regarding influence of its main constituents such as safranal, crocin and crocetin on the immune system cellularity and function. Since the SAF is widely used throughout the world, we decided to survey its effects on mice immune system using by immunotoxicity standard tests. Our results showed that SAF at three different doses did not produce any signiﬁcant change in spleen/blood cellularity, HA, DTH, proliferation response to PHA, INF-γ/IL-4 ratios, INF-γ and IL-4 production. Also, histopathologically, SAF did not induce significant adverse effect on spleen and bone marrow tissues. 

To better understand regarding saffron effects and its components on immune system a systematic search was performed based on information available in known international medical databases. In this way, there was a study showing a preventive effect for SAF on tracheal responses and serum cytokine, total NO (nitric oxide) and nitrite levels as well as increased Th1/Th2 balance in sensitized guinea pigs. Of course, in that study SAF had been administrated orally to ovalbumin sensitized guinea pigs at three different concentrations (Boskabady et al., 2014 ▶) and a shift toward Th1 had been interpreted from measures of serum cytokines whereas in our study animals (mice) were treated by SAF intraperitoneally and we determined cytokines produced by isolated mice splenocyte in presence of PHA. Also, we found one study conducted on effects of saffron extracts on human peripheral blood mononuclear cells (PBMC), in which saffron has partly shown immunomodulatory effects. In this study, the effects of extracts on cell viability and cytokine release of stimulated peripheral blood mononuclear cells by PHA and non-stimulated cells were examined. In cells stimulated with PHA, different concentrations of the extract showed a significant inhibition on cell viability of lymphocytes. Saffron extract at high level (500 μg/ml) was able to reduce IFN-γ production in stimulated cells and IL-10 secretion in both stimulated and non-stimulated cells. Saffron at all levels also showed a significant increased ratio of IFN-γ to IL-4 (Boskabady et al., 2011 ▶). In another study performed by Vijayabhargava and Asad that saffron had been orally administered as suspension at doses of 50 and 100 mg/kg, saffron extract significantly had increased the level of serum immunoglobulins and circulating antibody titer, prevented the mortality in mice when challenged with lethal Pasteurella multocida toxin. These findings indicate that saffron had been able to elevate the power of humoral mediated immunity. Saffron at low dose was effective in inducing a significant increase in the phagocytic activity. They concluded that *C. sativus* at low doses stimulate humoral and cell mediated immunity and it could have been considered as a potential immunostimulant and as an anticancer agent (Vijayabhargava and Asad, 2011 ▶). 

On the other hand, our results showed that SAF was safe to mice immune system. Perhaps, combination use of SAF with other constituents present in saffron extract such as crocin might have caused some reactions which were observed in Boskabady et al and Vijayabhargava and Asad studies. For example, such effects observed in two last mentioned studies might be more due to the anti-oxidant activity of SAF and crocin of saffron extract that might reinforce each other to bring saffron immunomodulatory effects, whereas in our study, SAF by itself was not able to induce these effects (Vaibhav et al., 2011 ▶, De la Fuente and Victor, 2000 ▶). 

In normal conditions, the reaction process of lymphocytes with antigens, lymphokines, or with other cell subsets needs the cell membrane integrity. It is possible that free oxygen radicals generated through normal function of immune cells could finally diminish the activation of immune cells via membrane lipids peroxidation. Therefore, saffron extract containing anti-oxidant ingredients might induce some immunomodulatory effects via scavenging the ROS produced by immune cells (Assimopoulou et al., 2005 ▶, Kanakis et al., 2007 ▶). 

In addition, toxicological studies have demonstrated that SAF is more toxic than other active components in saffron stigma. According to Iranian traditional medicine, it has been proved that administration of whole herbs shows more activity and fewer side effects than isolated constituents (Ziaee et al., 2014 ▶). For example, in a study performed by Ziaee and colleagues, it was showed that saffron reduced toxic effects of its components, SAF, in acute and subacute toxicities in rats. Therefore, in Boskabady et al and Vijayabhargava and Asad studies, the immunomodulation seen by saffron administration might have been due to usage of intact saffron stigma (Ziaee et al., 2014 ▶). So, perhaps, lack of immunodulatory effects in our work might be partly due to general toxicity effects of intact SAF. Of course, the doses of SAF used here were based on a study demonstrating the anti-nociceptive and anti-inflammatory effects of SAF at doses to mice of 0.1–0.5 ml/kg (Hosseinzadeh and Shariaty, 2007 ▶). 

In conclusion, SAF as a major constituent of saffron did not induce any marked effects in immune system parameters of mice in spite of a few studies demonstrating some immunomodulatory effects for saffron extract. Contrary to the researches that indicated the toxicity of SAF is more than that of other active constituents in saffron stigma, at least it was found to be safe to mice immune system and has no toxicity on humoral and cellular immune responses. 
